# A Successfully Treated Multiple Metastatic Choriocarcinoma Coexistent With Live Fetus: A Case Report and Literature Review

**DOI:** 10.3389/fonc.2021.777707

**Published:** 2022-01-31

**Authors:** Wei Ding, Na Zhang, Yang Rao, Xiaoning Xu, Tonggang Nie, Pengpeng Qu

**Affiliations:** ^1^ Tianjin Central Hospital of Gynecology Obstetrics, Tianjin Key Laboratory of Human Development and Reproductive Regulation, Tianjin, China; ^2^ Department of Gynecological Oncology, Tianjin Central Hospital of Gynecology and Obstetrics, Tianjin, China; ^3^ Institute of Forensic Science, Tianjin Municipal Public Security Bureau, Tianjin, China; ^4^ Clinical School of Obstetrics and Gynecology Center, Tianjin Medical University, Tianjin, China

**Keywords:** gestational choriocarcinoma, metastases, chemotherapy, short tandem repeat, case report, literature review

## Abstract

Management of metastatic choriocarcinoma coexistent with live fetus is tricky for gynecologists. There is no consensus on treatment because of its rarity. We present a unique case of gestational choriocarcinoma with multiple metastases, who received EP chemotherapy in the third trimester. At 31 + 5 weeks, a healthy male baby was delivered by cesarean section. Then, she received six cycles of EMA/CO as postpartum chemotherapy. Her beta-human chorionic gonadotropin (β-hCG) level decreased to the normal range, and the metastases vanished. The patient had no clinical symptoms 4 years after discharge, and the baby was also free from this disease. Short tandem repeat polymorphism (STR) analysis was performed to determine the genotype of the choriocarcinoma, placenta, and normal curettage tissue of the maternal uterine. Comparing the polymorphic genetic markers revealed that the tumor was gestational choriocarcinoma, but did not originate from the coexistent pregnancy. In spite of extensive metastases, antepartum chemotherapy is an effective and safe treatment for patients with gestational choriocarcinoma concurrent with pregnancy. STR analysis can be useful in distinguishing gestational choriocarcinoma from non-gestational, as well as the causative pregnancy, and serve as a helpful examination tool for guiding clinical management.

## Introduction

Choriocarcinoma is a highly aggressive trophoblastic malignancy with two subgroups: gestational and non-gestational. Most choriocarcinomas are gestational, originating from pregnancies, whether abnormal or normal. Non-gestational choriocarcinomas may arise as a germ cell tumor, most commonly as a component of a mixed germ cell tumor, or as somatic carcinomas, generally as a component of a poorly differentiated carcinoma or adenocarcinoma. Choriocarcinoma concurrent with pregnancy is extremely rare, with an estimated 1 in every 160,000 pregnancies, and most of the patients terminated pregnancy immediately (1). In this report, we described a rare case of metastatic choriocarcinoma with a coexistent live fetus. The patient received antepartum chemotherapy in the third trimester and then delivered a healthy infant by cesarean section. The choriocarcinoma tissue of the patient was analyzed by morphology and DNA marker analysis.

## Case Presentation

This case presentation has obtained the patient’s consent and the approval of the ethics committee of the Tianjin Central Hospital of Obstetrics and Gynecology, Tianjin, China. On May 10, 2017, a 29-year-old pregnant (gravida 4, para 2) at 25 + ^4^ gestational weeks was admitted, complaining of vaginal bleeding for 2 weeks. The patient recalled a spontaneously ceased vaginal bleeding and moderate hyperemesis during the first trimester, but neither of which was clinically treated. She underwent scheduled antenatal examination, and an ultrasound examination at 21 gestational weeks showed a normal fetus and a solid mass between the amniotic sac and anterior wall of the uterus measuring 4×1 cm, which was thought to be an obsolete hematocele. She had bed rest for a month, but vaginal bleeding occurred at 25 gestational weeks, and she visited a doctor at a local hospital. Her previous menstrual cycles were regular, and she had given birth to two female infants in spontaneous labor at term in 2009 and 2011, respectively. Her last pregnancy was a spontaneous miscarriage in July 2016.

**Figure 1:**
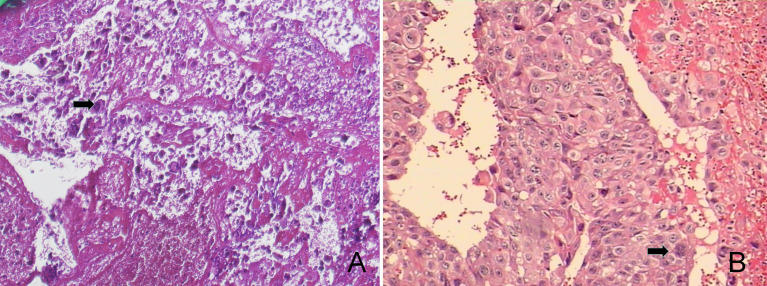
The pathological findings were choriocarcinoma accompanied with extensive hemorrhage and necrosis (100×, **A**). The tumor consists of syncytiotrophoblastic cells and cytotrophoblastic cells, which were characterized as medium-sized, whereas the peripheral one was composed of large, multinucleated cells (200×, **B**).

The patient was in generally good condition. The size of the uterus was consistent with the gestational age without uterine contractions. The fetal heart rate ranged from 135 to 155 beats per minute. A 4×3×3 cm purplish mass was found at the vaginal introitus, expelling necrotic tissue through superficial ulcers. A 2×1×0.5 cm nodule was found on the anterior vaginal wall. Microscopically, the necrotic tissue showed the plexiform pattern with triphasic differentiation into cytotrophoblast, syncytiotrophoblast, and intermediated trophoblast and marked cytologic atypia, confirmed as metastatic choriocarcinoma (**Figure 1**).

**Figure 2:**
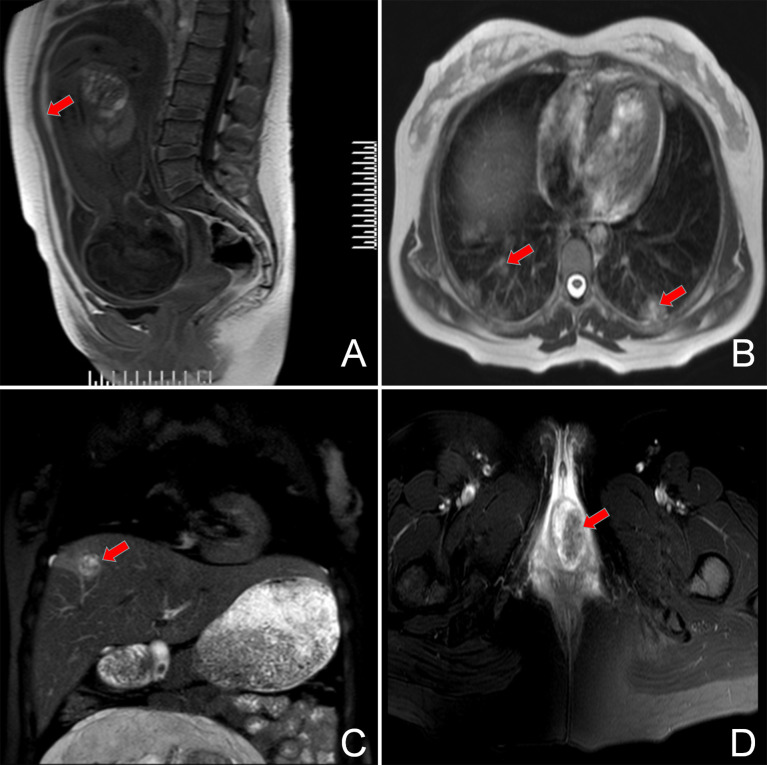
MRI film showing the original lesion in the anterior uterine wall **(A)** and the metastasis lesion in the bilateral pulmonary tissue **(B)**, right lobe of the liver **(C)**, and the vagina **(D)**.

The patient was referred to our department of gynecological oncology. A comprehensive examination was performed: color doppler ultrasonography showed a single intrauterine pregnancy at 28 gestational weeks, and dilation of the muscular vessels in the anterior uterine wall; chest computed tomography (CT) revealed more than 10 multiple lung metastases with the largest lesion about 3.0×1.9 cm; abdominal magnetic resonance (MR) presented a 3.2×2.1 cm hemorrhagic tumor in the right lobe of liver; pelvic MR revealed extensive hematocele between the anterior uterine wall and amniotic sac with the thickness of about 6 mm, and a 39×31×31 mm metastatic lesion in the vagina, without fetal metastasis; head MR revealed no brain metastasis; the level of serum β-hCG was 26,603 mIU/ml (normal <5 mIU/ml, range for gestational age of 29 weeks 940–60,000 mIU/ml) (**Figure 2**). With the combination of the clinical presentation and examination results, the patient was diagnosed as an intrauterine pregnancy complicated with choriocarcinoma (stage IV) with vaginal, liver, and pulmonary metastases. After proper counseling, the woman wanted to continue the pregnancy and receive antepartum chemotherapy despite of the oncological risk.

The patient started antepartum chemotherapy with EP regimen (etoposide 100 mg/m^2^ and cisplatin 20 mg/m^2^) once a week. Meanwhile, 6 mg of dexamethasone was administered intravenously every 12 h for 2 days to promote fetal lung maturation. Fetal heart monitoring was conducted weekly, and the falling of serum β-hCG level was remarked. After two cycles of intravenous EP chemotherapy, the vaginal metastases shrank gradually, and the β-hCG level decreased to 20,082 mIU/ml. At 31 + ^5^ gestational weeks, the patient complained of regular contractions and refused to spontaneous labor. A cesarean section was then performed. A healthy male infant was delivered, weighing 1,950 g with an Apgar score of 9 for the first minute. A stiff mass about 4×3×3 cm could be palpable on the endometrium in the anterior uterine wall, and bilateral ovaries and fallopian tubes were normal. The placenta and uterine curettage tissues were sent for histopathologic examination. The final pathology diagnosis was choriocarcinoma after chemotherapy. The infant was tested negative for serum β-hCG.

The genotype of the choriocarcinoma tumor tissue, placenta, and normal curettage tissue from the maternal uterus were analyzed using STR. DNA was extracted from paraffin-embedded material (QIAamp DNA FFPE Tissue Kit, CA, USA). The fragments were analyzed by capillary electrophoresis on an ABI 3500XL genetic analyzer (Applied Biosystems, CA, USA). We analyzed 21 microsatellite loci on 19 chromosomes (AMEL, CSF1P0, D1S1656, D13S317, D16S539, D18S51, D19S433, D21S11, D2S1338, D12S391, D3S1358, D5S818, D6S1043, D7S820, D8S1179, FGA, TH01, TPOX, vWA, Penta E, Penta D). Data were analyzed using GeneMarker v.3.2 (SoftGenetics, Pennsylvania, CA, USA).

**Figure 3:**
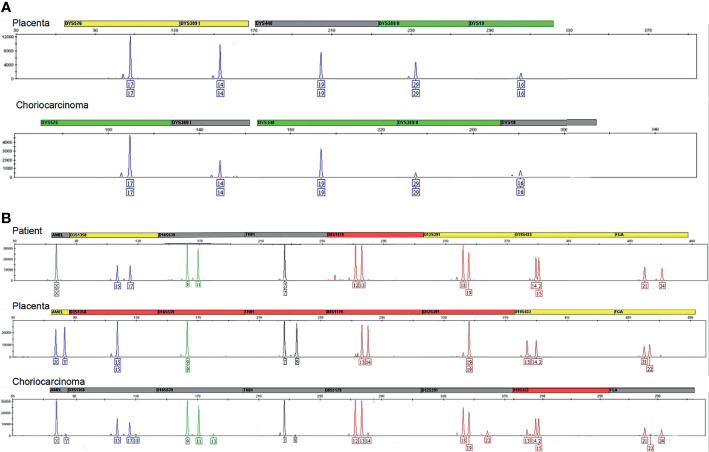
**(A)** Results for the different markers located on the chromosome Y. All markers included DYS576, DYS389I/II, DYS448, and DYS19 indicated the chromosome Y in the placenta and choriocarcinoma originated from one paternal genotype. **(B)** The markers D3S1358, D16S539, TH01, D8S1179, D12S391, D19S433, FGA indicated autosomal STR of choriocarcinoma was different from that of the placenta tissue.

According to the genotype, the choriocarcinoma showed a signal on Y-chromosome, which matched that of the placenta (**Figure 3A**). This result indicated the presence of paternal DNA in the tumor, suggesting the gestational origin of the choriocarcinoma. Since the patient delivered two healthy female infants in 2011 and 2012, the probable origin of the choriocarcinoma was either from the third spontaneous abortion or the fourth male infant. Because the third abortion tissue was not sent for pathological examination, it is a pity that the genotype could not be detected. However, according to the autosomal STR results, we found that the autosomal STRs of choriocarcinoma was different from that of the placenta tissue (D3S1358, D16S539, TH01, D8S1179, D12S391, D19S433, FGA, **Figure 3B**). Therefore, the choriocarcinoma tissue was not derived from the fourth child or the patient, and the third spontaneous abortion was speculated to be the origin.

**Figure 4:**
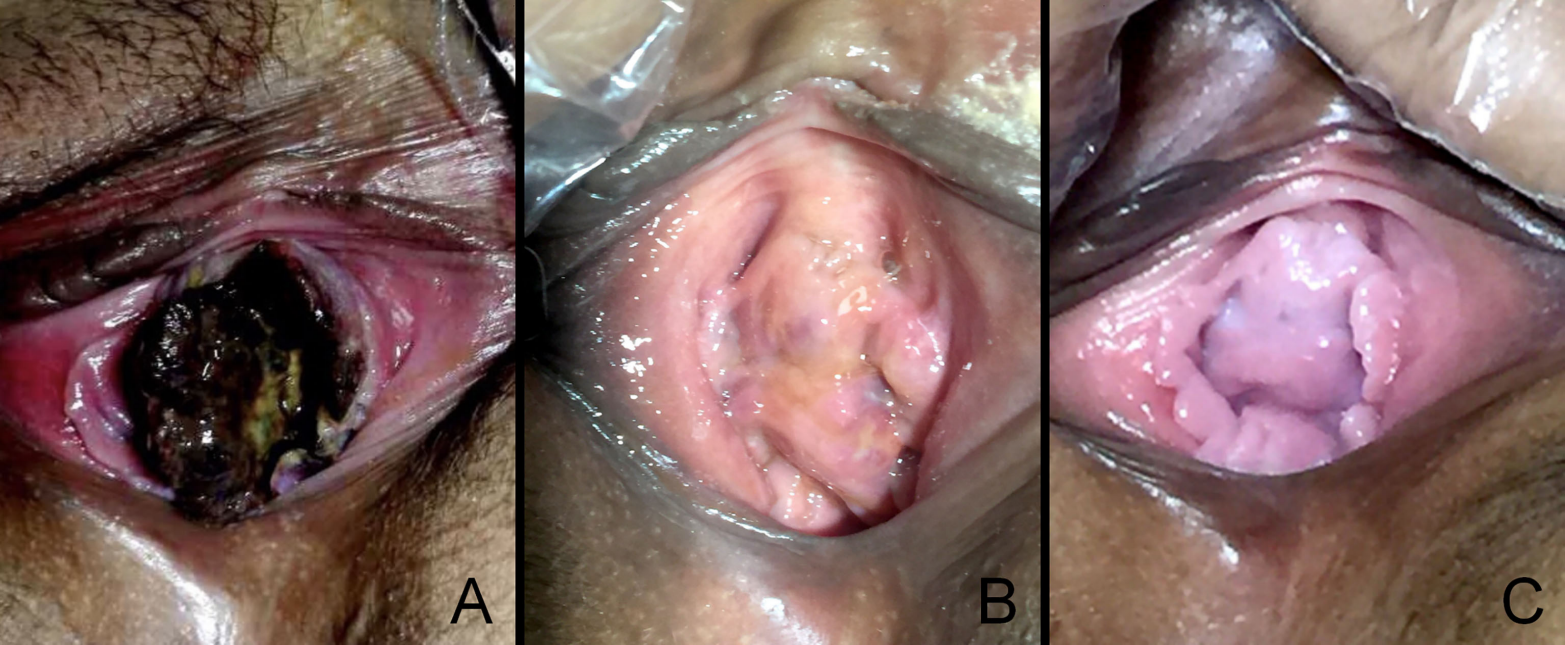
The vaginal metastasis choriocarcinoma, which contained blood clots and necrotic tissue before chemotherapy **(A)**, at 30 days postdelivery **(B)**, at 90 days postdelivery when finished with EMA/CO chemotherapy **(C)**.

Because the patient was gratified as high-risk, the chemotherapy regimen was upgraded to EMA/CO (etoposide 100 mg/m^2^, methotrexate 100 mg/m^2^, 200 mg/m^2^ with folic acid, actinomycin D 0.5 mg, cyclophosphamide 600 mg/m^2^, and vincristine 1 mg/m^2^). After three cycles, the β-hCG value was 0 mIU/ml, and vaginal metastases vanished completely (**Figure 4**). Subsequently, a thoracic CT and abdominal MR revealed a total regression of the metastatic lesions, and her menstruation recovered 7 months after chemotherapy.

The child was healthy, without evidence of related illness, and the mother had no clinical manifestations for 4 years after discharge. The serum β-hCG level was normal, and no metastasis was observed.

## Discussion

Choriocarcinoma is a highly aggressive, malignant trophoblastic neoplasm. Most of them are gestational in origin with a paternal component identified by DNA analysis, while non-gestational choriocarcinoma, which develops as a component of a germ cell tumor or is related to a somatic mutation of a poorly differentiated carcinoma, is extremely rare. Gestational choriocarcinoma may follow any pregnancy and is mostly associated with coincident or antecedent pregnancy, including miscarriage, term pregnancy, or molar pregnancy. Although gestational and non-gestational choriocarcinoma share similar pathological and morphological features, they were different in genetic origin, immunogenicity, sensitivity to chemotherapy, and prognosis. Genetically, gestational choriocarcinoma is considered to be an allograft, is more immunogenic, and responds well to chemotherapy, in contrast to non-gestational choriocarcinomas originating entirely from the patient. Gestational choriocarcinoma has a better prognosis than non-gestational choriocarcinoma (2). Chemotherapy is the main treatment option for gestational choriocarcinoma, while non-gestational choriocarcinoma is treated with surgery combined with chemotherapy as determined by disease stage. The incidence of choriocarcinoma coexisting with or after an otherwise normal, viable pregnancy is extremely rare, with about 1 in every 160,000 pregnancies (3). We searched the English literature in PubMed and found 18 patients who were diagnosed with metastatic choriocarcinoma coexistent with a viable pregnancy. Their data are listed in **Table 1**. The mean age of the patients was 28.7 years, and the mean gestational age at diagnosis was 26.5 weeks. The most common sites of metastases included lungs (89%), brain (39%), vagina (17%), and liver (11%) (4–20).

**Table 1:** Summary of widespread metastasis of choriocarcinoma during pregnancy.

Reference	Age	Gestational week at cancer diagnosis/chemotherapy exposure	Metastatic site	Gestational week of delivery	Type of delivery	Birth weight, g	Newborn’s sex	Chemotherapy	Stage
Nabers 1990 (4)	36	27/27	Lungs	34	NVD	2,000	Male	MTX	III
Gangadharan 1993 (5)	25	30/30	Lungs, vagina	32	NVD	NA	Male	MTX	III
Dana 1996 (6)	32	32/no	Brain, lungs	33	C/S	1,920	Female		IV
Zanetta 1997 (7)	37	7/no	Vagina	9	TA				II
Zanetta 1997	28	30/no	Lungs	32	C/S	NA	NA		III
Mamelak 2002 (8)	27	27/no	Brain	30	C/S	NA	NA		IV
Picone 2003 (9)	20	28/no	Brain, lungs	28	C/S	1,260	Female		IV
Lee 2005 (10)	31	32/no	Lungs	33	C/S	2,160	Male		III
Ahmed 2006 (11)	31	33/no	Lungs	NA	C/S	NA	NA		III
Bircher 2011 (12)	25	23/23	Lungs	25	NVD	709	Female	MTX	III
Brudie 2011 (13)	28	20/24	Lungs, liver, brain	32	NVD	1,383	Female	EMA/CO	IV
Zhu 2013 (14)	28	30/no	Vagina, lungs, brain	31	C/S	1,800	Female		IV
Stefano 2014 (15)	30	28/no	Lungs	31	C/S	1,263	Male		III
Kristiansen 2016 (16)	33	22/no	Lungs, liver	22	Hysterectomy/TA	NA	Female		IV
Yu 2016 (17)	29	32/no	Lungs, brain	33	C/S	1,800	Male		IV
Maeda 2018 (18)	33	31/no	Lungs	31	C/S	1,390	Female		III
Leticia 2020 (19)	22	31+6/no	Lungs	32+1	C/S	1,400	Female		III
Li 2021 (20)	21	14/no	Lungs, brain	34	C/S	1,590	Female		IV

NVD, normal vaginal delivery; C/S, caesarian section; NA, not applicable; TA, terminated; EMA/CO, etoposide, methotrexate, actinomycin D, cyclophosphamide, vincristine.

Although vaginal bleeding is usually the common clinical manifestation of choriocarcinoma, there are non-gynecological symptoms mostly caused by metastases. Metastases tend to occur in the lungs, causing respiratory symptoms like cough, hemoptysis, and dyspnea. Metastases occur less frequently in the brain, presenting neurological symptoms like headaches, sensory disturbance, rarely in the vagina, pelvis, liver, and lymph nodes (21–23). The patient in our study was admitted because of mild vaginal bleeding during the second trimester, and the pathological findings of vaginal mass confirmed the diagnosis of choriocarcinoma. In fact, the woman had a short-time vaginal bleeding in the first trimester, and an ultrasound scan found a solid mass between the amniotic sac and the uterus at 21 gestational weeks; however, these symptoms did not draw the doctor’s attention. Therefore, choriocarcinoma needs to be considered when abnormal bleeding and a mass in the uterus occurred at any time during pregnancy.

Since most patients with choriocarcinomas concurrent with pregnancy have been pregnant before, it is difficult to determine whether the choriocarcinoma is originated from the present pregnancy or their prior pregnancies. Therefore, for women with a history of childbirth, genetic analysis, including microsatellite polymorphism, locus-specific genetic markers, and Y-chromosome-specific fluorescence *in situ* hybridization probes, is required to distinguish non-gestational choriocarcinoma or gestational choriocarcinoma, and to determine which pregnancy was the origin. However, genetic analysis is lacking in most reported cases. Kanehira reported one case of intraplacental choriocarcinoma was originated from normal gestation. The genotype of all loci from the tumor matched the placenta by the polymorphic genetic analysis, indicating the transition of normal chorionic villi to the malignant proliferation of trophoblast (24). According to the Y-chromosome and autosomal STR results, we speculated that the choriocarcinoma was originated from spontaneous abortion.

Gestational choriocarcinoma is sensitive to chemotherapy drugs, so once diagnosed, chemotherapy is often the main treatment, with surgery and radiation as an adjunct. The patients with FIGO prognostic score ≤6 were treated with a single-drug chemotherapy regimen and those with FIGO stage II, III disease and a prognostic score ≥7, or FIGO stage IV should be treated with multi-agent chemotherapy (25). However, for patients with choriocarcinoma concurrent with pregnancy, chemotherapy will increase the rate of fetal malformation and spontaneous abortion during 2 to 8 gestational weeks, and chemotherapy also increases the risk of bleeding and infection during childbirth. In the third trimester of pregnancy, the impact on the fetus is less. The chemotherapy is recommended to be used 3 to 4 weeks before pregnancy termination to control the progress of cancer and improve the survival rate by waiting for the fetus to mature (26). In addition, Charing Cross Hospital strongly recommends that for gestational trophoblastic neoplasia patients with extensive metastases, high disease burden, or FIGO scores ≥ 12, direct administration of standard first-line chemotherapy EMA/CO may cause hemorrhage and even multiple organ failure. To avoid these serious adverse reactions, the center used a low-dose EP regimen with two to three cycles of induction as initial chemotherapy, and this method reduced the early death rate from 7.2% to 0.7% (27). Considering the disease stage IV and the prognostic score of 14 (age: 0, antecedent pregnancy: 1, interval: 2, hCG: 2, number of metastases: 4, site of metastases: 4, largest tumor mass: 1, prior chemotherapy: 0) of this patient, we chose two cycles of intensive platinum-based single-agent therapy. EMA/CO combination chemotherapy was offered immediately after birth, and a satisfactory effect was achieved. In our review, the cases described by Nabers (4), Gangadharan (5), Bircher (12), and Brudie (13) received antepartum chemotherapy, and live fetuses were delivered after chemotherapy. In the case described by Brudie (13), the treatment was started at the 24^th^ week of gestation with EMA/CO; in the other three cases, at the 23^rd^ (12), 27^th^ (4), and 30^th^ (5) week with MTX, respectively. Three infants did not show significant handicaps or malformation, one infant was deaf, but it is unclear whether this is a result of her prematurity or the chemotherapy (MTX) her mother received during her pregnancy. One patient underwent hysterectomy at 22 weeks, and the female fetus died within an hour (5). Nine patients chose a cesarean section to terminate their pregnancies immediately after the diagnosis. Four patients chose to observe for a period until delivery. The outcomes of most mothers are usually good. The data of the newborns and their long-term follow-up were incomplete. It was highly related to the week of gestation and the availability of adequate neonatal care. To sum up, it is relatively safe to perform chemotherapy during the second and third trimester. However, additional research is needed to explore the safety and effectiveness of different chemotherapy.

## Conclusion

Although choriocarcinomas concurrent with pregnancy are rare, clinicians should consider this disease when abnormal bleeding and a mass in the uterus occurred during pregnancy. It is relatively safe to perform chemotherapy during the second and third trimester. The determination of the origin of the choriocarcinoma is important for treatment option and prognosis assessment. STR analysis can be useful in distinguishing gestational choriocarcinoma from non-gestational, as well as the causative pregnancy, and serve as a helpful examination tool for guiding clinical management.

## Data Availability Statement

The raw data supporting the conclusions of this article will be made available by the authors without undue reservation.

## Ethics Statement

The studies involving human participants were reviewed and approved by the institutional research ethics committee of Tianjin Central Hospital of Obstetrics and Gynecology. The patients/participants provided their written informed consent to participate in this study. Written informed consent was obtained from the individual(s) for the publication of any potentially identifiable images or data included in this article.

## Author Contributions

PQ and WD designed and organized the study. XX and TN were responsible for the genetic counseling of the patient. NZ and YR collected and prepared the archived choriocarcinoma tissue. WD wrote the first article draft. PQ supervised the study. All authors contributed to the article and approved the submitted version.

## Funding

Tianjin Municipal Health Commission (no. KJ20098) and the Open Fund of Tianjin Central Hospital of Gynecology Obstetrics/Tianjin Key Laboratory of Human Development and Reproductive Rregulation (2019XH08).

## Conflict of Interest

The authors declare that the research was conducted in the absence of any commercial or financial relationships that could be construed as a potential conflict of interest.

## Publisher’s Note

All claims expressed in this article are solely those of the authors and do not necessarily represent those of their affiliated organizations, or those of the publisher, the editors and the reviewers. Any product that may be evaluated in this article, or claim that may be made by its manufacturer, is not guaranteed or endorsed by the publisher.

## Supplementary Material

Supplementary Figure 1 Y chromosomal STR data in placenta.

Supplementary Figure 2 Y chromosomal STR data in choriocarcinoma.

Supplementary Figure 3 Autosomal STR data in uterus.

Supplementary Figure 4 Autosomal STR data in placenta.

Supplementary Figure 5 Autosomal STR data in choriocarcinoma.
