# Supplementary figures and images for: A Successfully Treated Multiple Metastatic Choriocarcinoma Coexistent With Live Fetus: A Case Report and Literature Review
Source: Front Oncol. 2022 Jan 31;11:777707. doi: 10.3389/fonc.2021.777707 (PMC8841587; doi:10.3389/fonc.2021.777707)

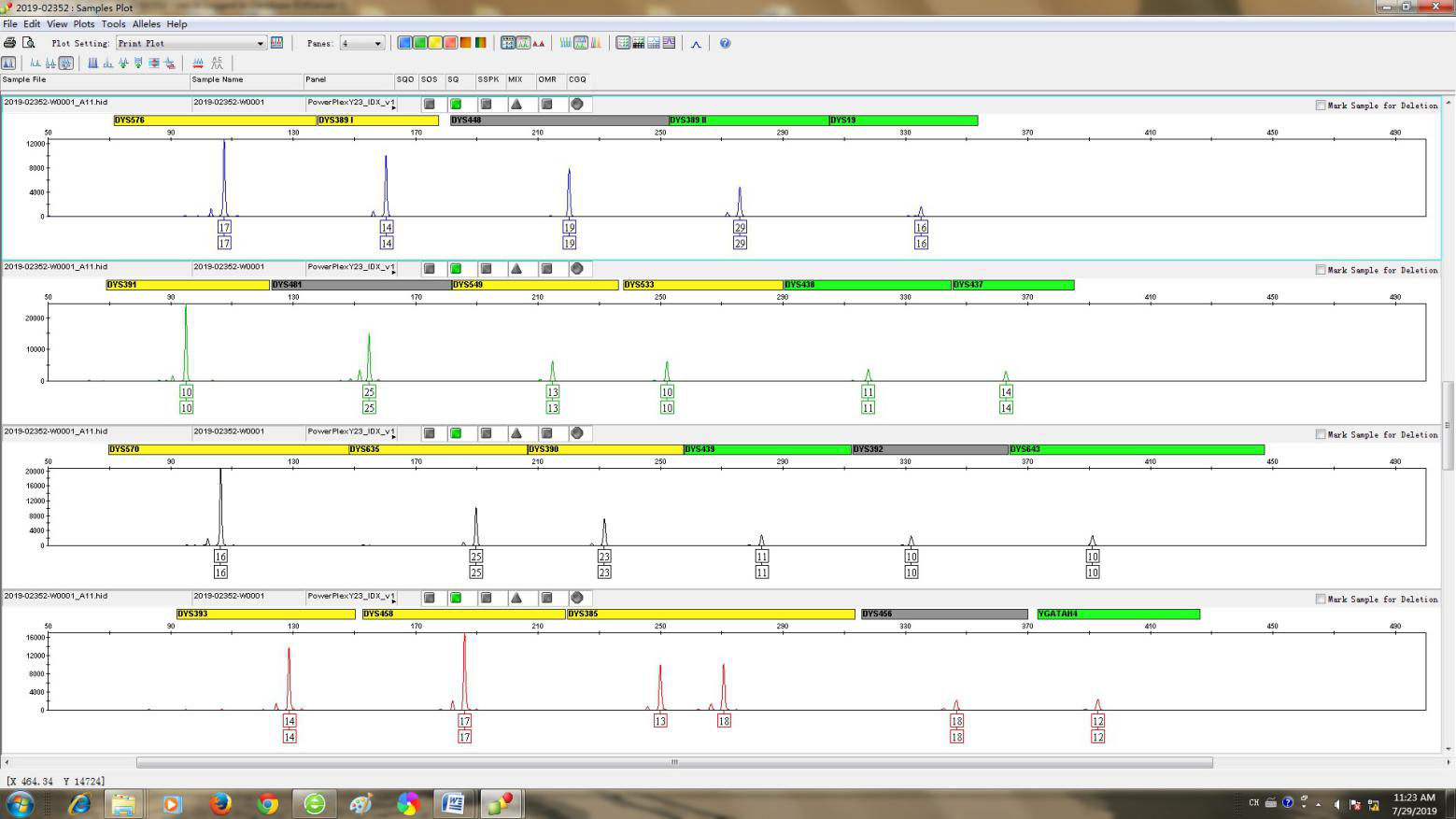

Supplement: Supplementary Figure 1 — Y chromosomal STR data in placenta. [file Image_1.tif]

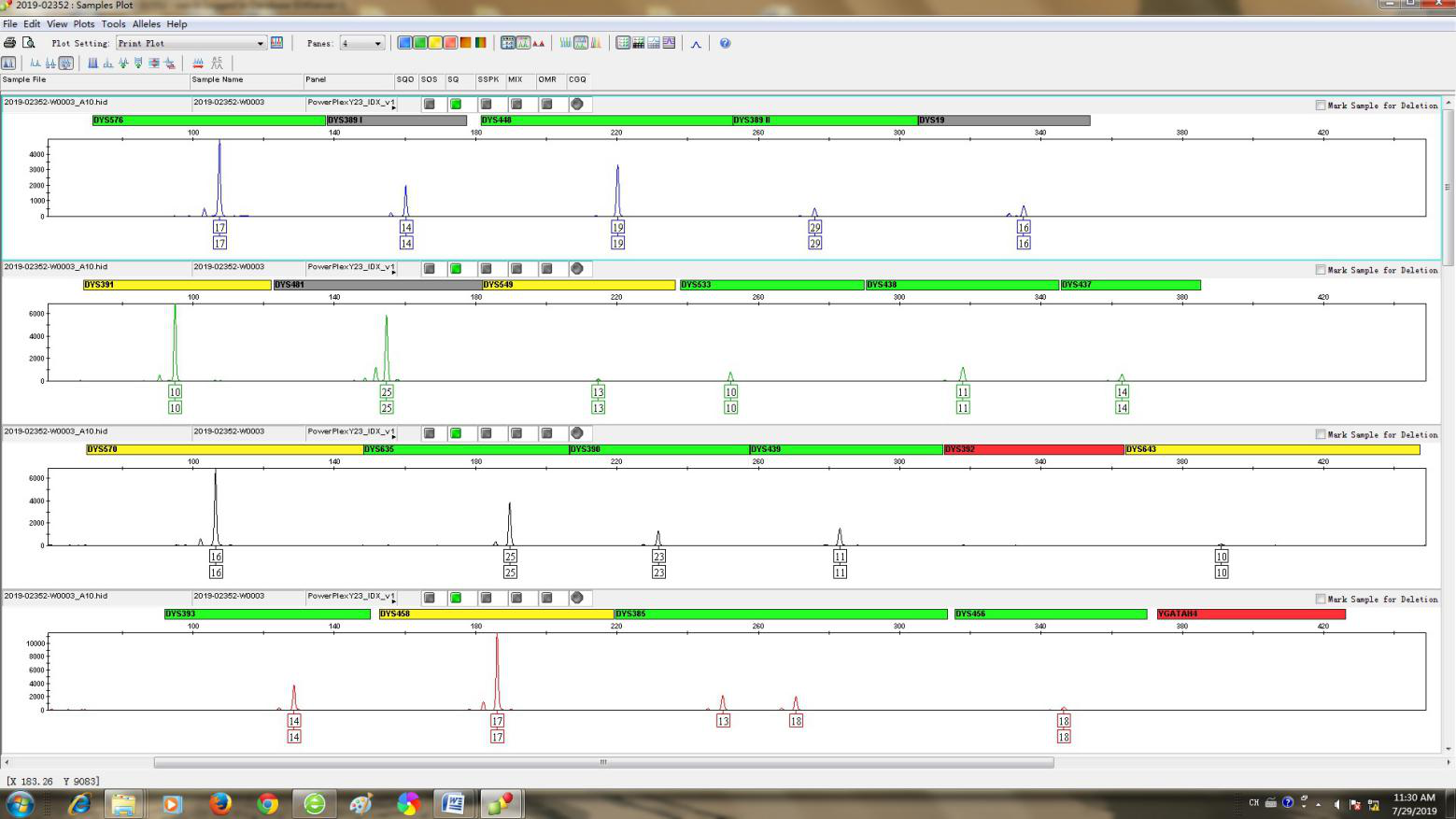

Supplement: Supplementary Figure 2 — Y chromosomal STR data in choriocarcinoma. [file Image_2.tif]

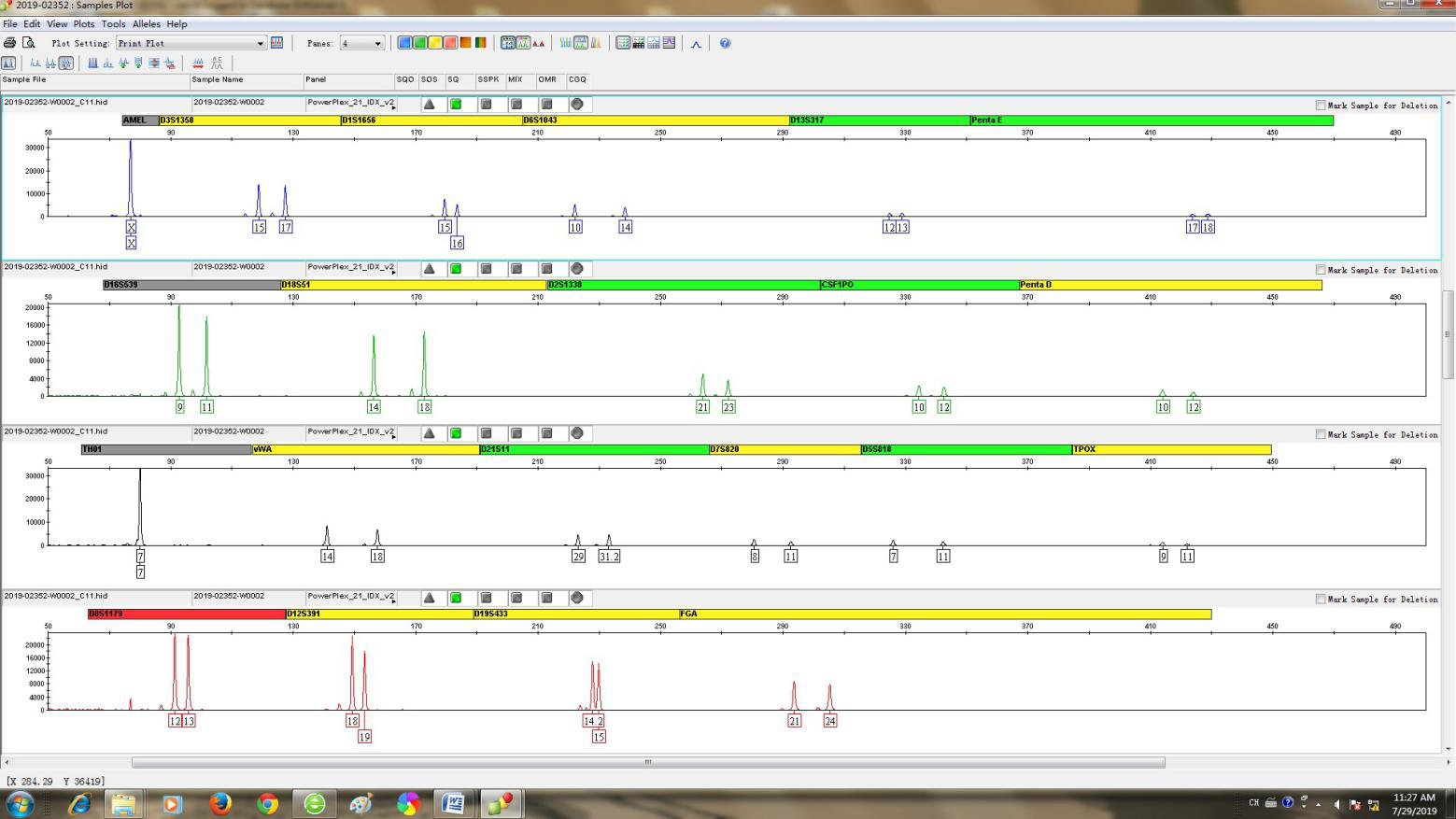

Supplement: Supplementary Figure 3 — Autosomal STR data in uterus. [file Image_3.tif]

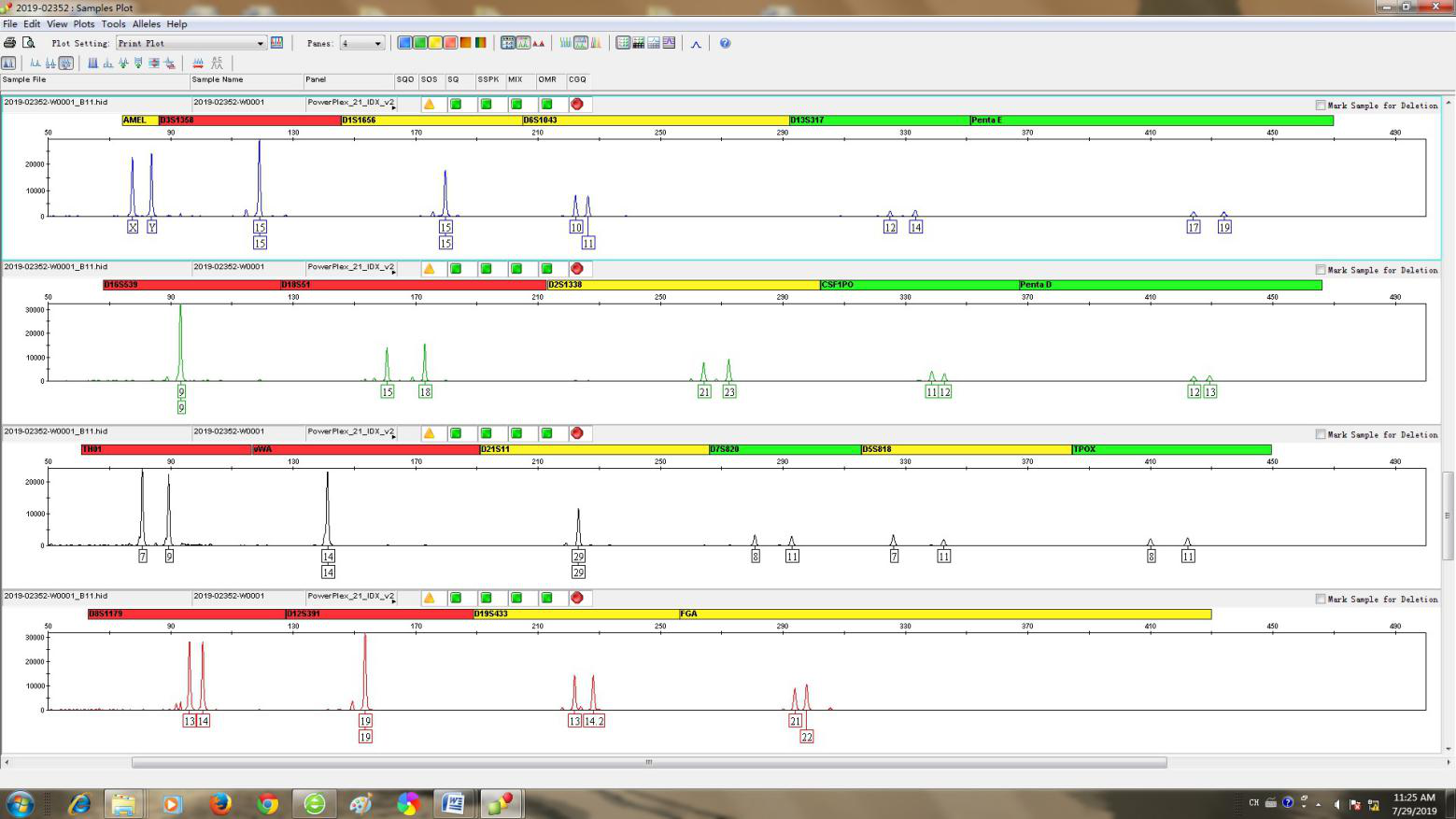

Supplement: Supplementary Figure 4 — Autosomal STR data in placenta. [file Image_4.tif]

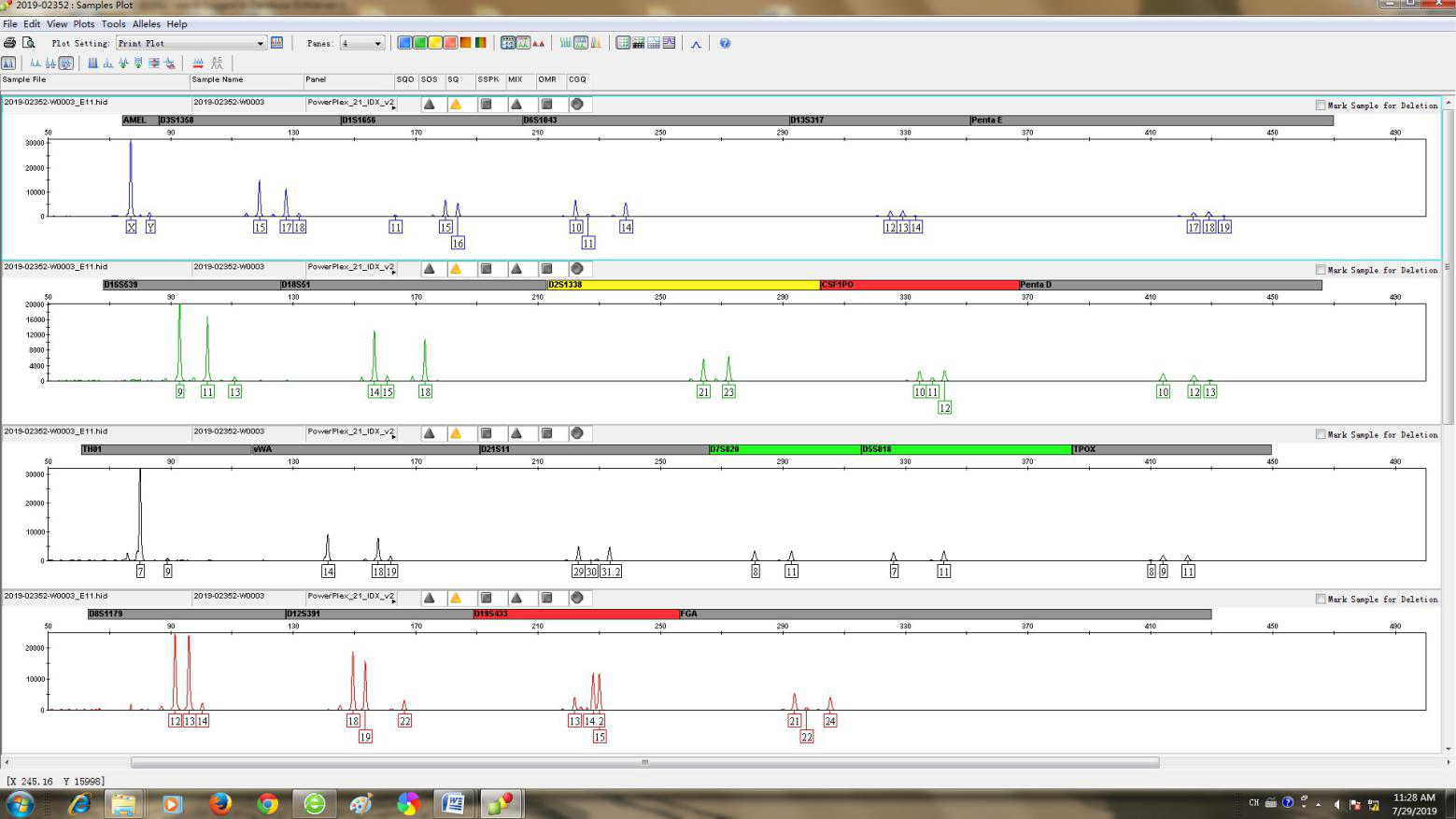

Supplement: Supplementary Figure 5 — Autosomal STR data in choriocarcinoma. [file Image_5.tif]
